# Traditional African Dishes Prepared From Local Biofortified Varieties of Pearl Millet: Acceptability and Potential Contribution to Iron and Zinc Intakes of Burkinabe Young Children

**DOI:** 10.3389/fnut.2019.00115

**Published:** 2019-08-14

**Authors:** Fatoumata Hama-Ba, Claire Mouquet-Rivier, Bréhima Diawara, Eva Weltzien, Christèle Icard-Vernière

**Affiliations:** ^1^Département de Technologie Alimentaire, IRSAT, Ouagadougou, Burkina Faso; ^2^Nutripass, IRD, University Montpellier, Montpellier, France; ^3^Honorary Fellow, Agronomy Department, University of Wisconsin-Madison, Madison, AL, United States

**Keywords:** biofortification, micronutrient, phytate, cereal, traditional dishes

## Abstract

Biofortification is among the food-based strategies, recently implemented and still in development, to fight micronutrient deficiencies. Three cereal-based traditional dishes of Sub-Saharan Africa (tô paste, pancakes, and gruel) prepared from one local (Gampela), or two biofortified (GB 8735 and Tabi) varieties of millet were assessed for their (i) acceptability by local consumers, (ii) iron and zinc absorption predicted by phytate-to-mineral molar ratios and (iii) contribution to the iron and zinc requirements of young children. Tasters preferred the color, texture, and taste of dishes prepared with the local variety, whether or not the grains were decorticated. Hedonic and preference tests showed no significant difference between the two biofortified varieties, but the cooks reported different behaviors during processing. Biofortified millet contained up to two times more iron than the local variety, reaching 6.5 mg iron/100 g dry matter. Iron and zinc contents remained higher in biofortified varieties even after decortication. Iron content in the dishes was highly variable, depending on iron loss and potential contamination during processing. The phytate-to-mineral molar ratios of all dishes indicated low iron absorption, independent of the millet variety, but improved zinc absorption in dishes prepared with biofortified varieties. The contribution of a dish prepared with one of the two biofortified millet varieties to the recommended iron and zinc intakes for 6–11-month-old children was estimated to be about 5 and 7%, respectively, compared to 2 and 4% for the same dish prepared with local millet. For 12–23-month-old children, the contribution to the recommended intakes was estimated to be about 14 and 12% with biofortified millet, respectively, and about 6 and 7% with local millet. The use of biofortified millet varieties could be complementary to food diversification strategies to increase iron and zinc intakes. As in Ouagadougou, cereals are eaten in different forms by young children several times per day, iron and zinc intakes could be improved in the long term by using the biofortified varieties of pearl millet.

## Introduction

About two billion people (1) worldwide were affected by micronutrient malnutrition like iron, vitamin A or iodine deficiencies, mainly in rural areas of developing countries (2). These data are still used as a reference because of the lack of more recent reliable data. In 2011, in Africa, 20.2% of children under 5 years of age and 20.3% of pregnant women had iron deficiency anemia, and 23.9% of the whole population had zinc deficiency (3). The diet of people in sub-Saharan Africa is based on staple foods (mainly cereals, but also legumes, roots, and tubers) that supply most of the energy requirements, but are often low in micronutrients, even more after processing. Micronutrients are found in greater amounts in vegetables, fruits, and animal-source foods. Cereals contribute little to meeting individual micronutrient requirements due to their low content in most minerals, and their high content in chelating factors, such as phytates and/or polyphenols that can reduce mineral bioavailability (4). These chelating compounds form insoluble complexes with divalent mineral cations, particularly iron and zinc, that cannot be absorbed in the duodenum. Processing of food can in some cases reduce the contents of chelating compounds and thus improve the bioavailability of minerals (5–8).

To address micronutrient deficiencies, traditional food-based strategies, such as fortification, biofortification, and food diversification, are combined with public health interventions (education, disease control, etc.) (9). Food fortification is a cost-effective strategy, but the availability of fortified foods in remote areas is difficult to ensure, and for iron, it is associated with technical problems mainly related to the choice of an iron compound suited to the chosen food vehicle. Food diversification, which involves increasing the consumption of items from micronutrient-rich food groups, is optimal in terms of sustainability, but sometimes hard to generalize due to limited food availability in some areas (9). Moreover, foods that are good sources of bioavailable iron or zinc are scarce. Indeed, these are mainly animal-source foods that are not available in sufficient quantities, or are too expensive for the poorest populations. Biofortification refers to techniques aiming at increasing the density of some vitamins or minerals in a staple food crop through plant breeding or agronomic practices (10) in order to enrich the edible portion of these micronutrients (11, 12). This is a complementary strategy to dietary diversification, particularly for those micronutrients which are poorly available in commonly consumed foods. After several years of development, vitamin A, zinc and/or iron biofortified crops have been released all over the world by consortiums, such as Harvest Plus, and many others are currently tested (13, 14). A review of public health strategies based on staple crop biofortification was even undertaken by specialized working groups, including technical and acceptability aspects, as well as public health considerations (15). As underlined by Meija and Boukerdenna (14), there is no need of specific standards and regulations concerning the food quality of products prepared with biofortified crops produced by conventional breeding, differently from those required for products obtained from genetic modification or nutrient-enhanced fertilization. Biofortified staples are often well-accepted by producers and consumers (16), when the biofortified trait is visible, as in the case of provitamin A biofortification that gives a yellow-orange color to the biofortified crops (17, 18), and also when visually undetectable, as is the case for iron (19) or zinc biofortified staple crops (20, 21). But acceptance studies of new biofortified crops are necessary before disseminating them or including them in feeding programs, as for beans in Colombia (22) or for pearl millet in India (21). The most important factors related to acceptance and adoption of biofortified crops in low- and middle-income countries were recently reviewed by Talsma et al. (23). They were shown to be context specific, and influenced by the color of the products, the season to which they are available and the nutritional information given. Biofortified crops must also prove their health benefits. Several efficacy and effectiveness studies showed the positive effects of biofortification on the micronutrient status of individuals (24, 25). However, some bottlenecks remain. For instance, the content of chelating factors, such as phytates, sometimes increases simultaneously with the level of iron in biofortified crops (26–28), thus impairing iron bioavailability and limiting the biofortification benefits. Bioavailability is also influenced by food processing (25, 29). Indeed, we previously showed that, due to partial removal of chelating factors during decortication, iron bioavailability in two decorticated biofortified millet varieties was slightly higher and zinc bioavailability was significantly higher than in the local unfortified variety (26).

Traditional African dishes are generally little studied in their “as eaten” form because most studies investigate grains or simply cooked staples. The objectives of this study were to (i) assess the acceptability, by usual consumers from Ouagadougou (Burkina Faso), of traditional dishes (tô paste, pancakes, and gruel) prepared with two biofortified varieties of millet compared with the same dishes prepared with a commonly used not biofortified local variety; (ii) analyse the changes in iron, zinc and phytate contents during processing of these traditional dishes, and calculate the predicted absorption values; and (iii) assess their potential contribution to the iron and zinc requirements of young children.

## Materials and Methods

### Plant Material

Three pearl millet (Pennisetum glaucum [L.]R.Br.) varieties were used. The two biofortified millet varieties, Tabi and GB 8735, were supplied by the International Crops Research Institute for the Semi-Arid Tropics (ICRISAT) in Mali. The GB 8735 variety, gray-colored, was obtained in 1987 by recurrent selection of local cultivars from Benin, Ghana, and Niger. The yellow Tabi variety is an open-pollinated variety recently introduced in Niger from northern Mali and is still undergoing performance trials. It is registered in the National Variety catalog of Niger. The local millet variety, called Gampela, was purchased from local producers working with the Environmental and Agricultural National Research Institute (INERA) in Ouagadougou (Burkina Faso).

### Traditional Dishes

For this study, three millet-based dishes that are among the most frequently consumed by young children in Ouagadougou were chosen (30): tô paste, pancakes, and fermented gruel. Tô is a thick paste obtained by cooking whole or decorticated grain flour with water, and is usually consumed together with a sauce, prepared from vegetables or green leafy vegetables. Pancakes are prepared by frying a fermented millet paste obtained from whole or decorticated grains. Gruels are prepared by fermenting a paste obtained by soaking whole millet grains followed by milling and wet sieving (31). Except for the gruel that was prepared only from whole grains, the other dishes were prepared with whole and with decorticated grains by nine Burkinabé women who were recruited for this study. They prepared the dishes in their traditional production unit or at home, using their own recipes and cooking utensils: aluminum pots, metal sieves, wooden calabash, plastic pots, and wood fires. Two-kg batches of whole and decorticated grains of each millet variety from the same lot were provided to the cooks. Millet grains were mechanically decorticated in the same decortication unit, as described previously (32). Whole and decorticated grains were then milled into flour by the women in their usual milling unit. The preparation of the final dishes was completed in the morning of the acceptability tests. The cooks' opinions about the processing features of the different varieties were collected on the same day.

### Analytical Methods and Absorption Prediction by Using the Phytate-to-Mineral Molar Ratios

Analyses were performed on raw and decorticated grains and freeze-dried samples of all prepared dishes.

*Dry matter* (DM) contents were determined using samples of 5–10 g by weighing after oven-drying at 105°C to constant weight.

*Phytate* content was determined after extraction of 0.2 g sample in 10 mL of 0.5M HCl at 100°C for 6 min. Phytate (in the form of myo-inositol hexaphosphate, IP6) content was measured by high performance anion-exchange chromatography using an AS-11 pre-column and column kit (Dionex, Sunnyvale, USA), as described previously (26). The separation was performed by gradient elution using NaOH 0.2 M solution and deionized water as eluents.

*Iron and zinc* were extracted with a closed-vessel microwave digestion system (Ethos-1, Milestone, Italy) from about 0.4 g of sample in a 7:1 nitric acid/hydrogen peroxide mixture. The closed vessels were placed in the microwave oven and digested at 1,200 W power for 30 min. Iron and zinc contents were analyzed with a Perkin-Elmer Analyst 800 atomic absorption spectrometer with a deuterium background corrector. The elements were identified by air-acetylene flame (32).

*Iron and zinc absorptions* were estimated by calculating the phytate-to-iron and phytate-to-zinc molar ratios, after conversion of the contents of minerals and phytates into moles. Iron absorption is improved when the phytate-to-iron molar ratio is lower than 1 and preferably lower than 0.4 in plain cereal and legume-based meals that do not contain iron absorption enhancers, such as ascorbic acid or meat (4). Zinc absorption is estimated to be low when the phytate-to-zinc molar ratio is higher than 18, and moderate for phytate-to-zinc molar ratios between 4 and 18 (33).

### Acceptability Tests

Acceptability tests were done at the Sensory Analysis Laboratory of IRSAT-DTA (Ouagadougou). Twenty-four adults, 12 women, and 12 men, who usually consume these millet-based dishes, were recruited on a voluntary basis. No personal information about participants was collected. Each of them was given a number that was used during acceptability tests and for further data analysis. Hedonic and preference tests were used to characterize the acceptability of the three dishes prepared with whole or decorticated, local or biofortified millet.

For the *hedonic tests*, the dishes were proposed individually to the tasters who successively evaluated their color, texture and taste, using a five level scale: very pleasant, pleasant, neither pleasant nor unpleasant, unpleasant, or very unpleasant.

For the *preference tests*, tasters had to rank the three millet-based dishes prepared with the local and the biofortified varieties from 1 (the sample the most appreciated) to 3 (the sample the less appreciated). Tasters were previously informed that the objective of the study was to compare dishes prepared with local and biofortified millet varieties obtained by conventional breeding and selected on the basis of their higher micronutrient content. However, tasters did not know how to recognize which dishes were prepared with biofortified varieties. Each participant tasted each of the three dishes prepared with the three millet varieties by three different producers on three successive days. Tests were conducted between 9:00 and 11:00 a.m. using dishes collected from the cooks on the morning of the test.

### Statistical Analysis

The hedonic test results were converted into scores ranging from 1 (most appreciated) to 5 (least appreciated), and compared with ANOVA and then Fischer's least significant difference test. For comparing the preference test results, the Friedman test was used, followed where appropriate, by rank sum multiple comparison tests. Statistical analyses were performed with the Statgraphics Plus software, version 5.1. In all comparisons, differences were considered statistically significant at *p* < 0.05.

## Results and Discussion

### Sensory Evaluation of Dishes Prepared With Local and Biofortified Varieties

The mean hedonic scores for color, texture, and taste of dishes prepared with biofortified millet ranged between 3 (neither pleasant nor unpleasant) and 4 (pleasant) (Table 1). However, tasters significantly preferred the color, texture, and taste of dishes prepared with the local variety Gampela whether they were prepared with whole or decorticated grains. Overall, dishes prepared with the biofortified variety Tabi received higher scores than those prepared with the other biofortified variety (GB 8735), but these differences were mostly not significant. Taste scores were significantly lower only for the tô prepared using whole GB 8,735 grains compared with the Tabi variety. Conversely, pancakes prepared from whole GB 8,735 millet had higher color, texture, and taste scores and were preferred to those prepared from whole Tabi grains (*p* < 0.05). For dishes prepared with decorticated millet, these differences were no longer significant.

**Table 1:** Results of the hedonic and preference tests for the three dishes prepared using local and biofortified millet varieties (*n* = 24*).

**Variety**	**Hedonic tests (mean score/5)**			**Preference test(rank)**
	**Color**	**Texture**	**Taste**	
**WHOLE MILLET**				
**Tô**				
Local Gampela	4.4^a^	4.4^a^	4.3^a^	1^a^
Biofortified Tabi	3.3^b^	3.5^b^	3.6^b^	2^b^
Biofortified GB 8735	3.1^b^	3.4^b^	3.3^c^	3^b^
*p*	<0.01	<0.01	<0.01	<0.05
**Pancakes**				
Local Gampela	4.6^a^	4.4^a^	4.2^a^	1^a^
Biofortified Tabi	3.1^c^	3.3^c^	3.5^b^	3^c^
Biofortified GB 8735	3.5^b^	3.8^b^	4.0^a^	2^b^
*p*	<0.01	<0.01	<0.01	<0.05
**Gruel**				
Local Gampela	4.6^a^	4.2^a^	4.2^a^	1^a^
Biofortified Tabi	3.7^b^	3.8^b^	3.8^b^	3^b^
Biofortified GB 8735	3.5^b^	3.6^b^	3.8^b^	2^b^
*p*	<0.01	<0.01	<0.05	<0.05
**DECORTICATED MILLET**				
**Tô**				
Local Gampela	4.7^a^	4.4^a^	4.5^a^	1^a^
Biofortified Tabi	3.4^b^	3.6^b^	3.4^b^	2^b^
Biofortified GB 8735	3.2^b^	3.3^b^	3.1^b^	3^b^
*p*	<0.01	<0.01	<0.01	<0.05
**Pancakes**				
Local Gampela	4.6^a^	4.1^a^	4.2^a^	1^a^
Biofortified Tabi	3.6^b^	3.7^b^	3.7^b^	2^b^
Biofortified GB 8735	3.3^b^	3.6^b^	3.8^b^	3^b^
*p*	<0.01	<0.01	<0.01	<0.05

*Data were compared with the ANOVA and Fischer's least significant difference tests (hedonic test scores), and the Friedman and rank sum multiple comparison tests to compare means for the preference test. Values in a column for the same dish with different superscript letters are significantly different (ANOVA; p < 0.05)*.

* *24 participants (12 women, 12 men) participated in the study. Each participant tasted each of the three dishes prepared with the three millet varieties by three different producers on three successive days*.

Overall, the acceptability of dishes prepared with biofortified varieties was good. The biggest differences with the local Gampela variety concerned the color of tô and gruels prepared with whole or decorticated GB8735 millet grains that are darker and thus resulted in dark products, moderately appreciated by tasters. However, this could be alleviated depending on the type of dish prepared, as shown by the good grades obtained by the pancakes and with processing adjustments. The cooks reported different processing behaviors for the three varieties. Biofortified GB 8,735 millet grains were harder than those of the other varieties, and consequently they needed longer soaking and cooking time for pancake and gruel preparation. However, all cooks appreciated the final taste and smell of the dishes prepared with this biofortified variety. The cooks also reported that biofortified Tabi millet underwent rapid swelling during tô and gruel cooking and that more oil was required for the preparation of pancakes with this millet compared with the other varieties due to higher absorption. Biofortified millet varieties could be promoted in the future together with appropriate information to producers and consumers, if they proved to increase iron and zinc intakes in the same conditions of consumption. In Mali, a recent study showed the high acceptability of whole grain foods prepared from biofortified sorghum varieties, based on information about its improved iron content (34). In India, acceptability of iron and zinc biofortified pearl millet, despite lower scores at hedonic tests compared to conventional pearl millet, was estimated as good enough to further integrate these biofortified varieties into an efficacy study (21).

### Iron, Zinc, and Phytate Contents of Local and Biofortified Millet Grains and Dishes

Iron and zinc contents in the whole biofortified millet varieties were two to three times higher than in the local variety (Table 2). The phytate content of the biofortified millet varieties was slightly higher than that of the local variety, as reported in other studies (28). After decortication, the iron, zinc and phytate contents of all three varieties were reduced, except for phytates in GB 8,735 millet. Indeed, as the grains of this biofortified variety were harder, they exhibited a higher decortication yield (26). This was appreciated by the cooks, but could also lead to lower losses in phytates. The iron and zinc contents remained higher in decorticated biofortified varieties than in the decorticated local variety.

**Table 2:** Iron, zinc, and phytate contents of local and biofortified millet varieties (*n* = 3).

**Millet**	**Whole grains**			**Decorticated grains**		
	**Iron (mg)**	**Zinc (mg)**	**Phytate (g)**	**Iron (mg)**	**Zinc (mg)**	**Phytate (g)**
	**For 100 g DM**					
Local Gampela	2.9 ± 0.0^a^	2.2 ± 0.0^a^	0.96 ± 0.01^a^	2.3 ± 0.2^a^	1.6 ±0.1^a^	0.85 ± 0.08^a^
Biofortified Tabi	5.3 ± 0.1^b^	3.6 ± 0.1^b^	1.08 ± 0.01^b^	3.9± 0.1^b^	2.8 ± 0.0^b^	0.83 ± 0.01^a^
Biofortified GB 8735	6.5 ± 0.0^c^	4.0 ± 0.2^b^	1.00 ± 0.01^b^	5.7 ± 0.3^c^	3.5 ± 0.0^c^	1.02 ± 0.01^b^

*Results are the mean ± standard deviation of three measurements; values in a column with different superscript letters are significantly different (ANOVA; p < 0.05)*.

**Figure 1:**
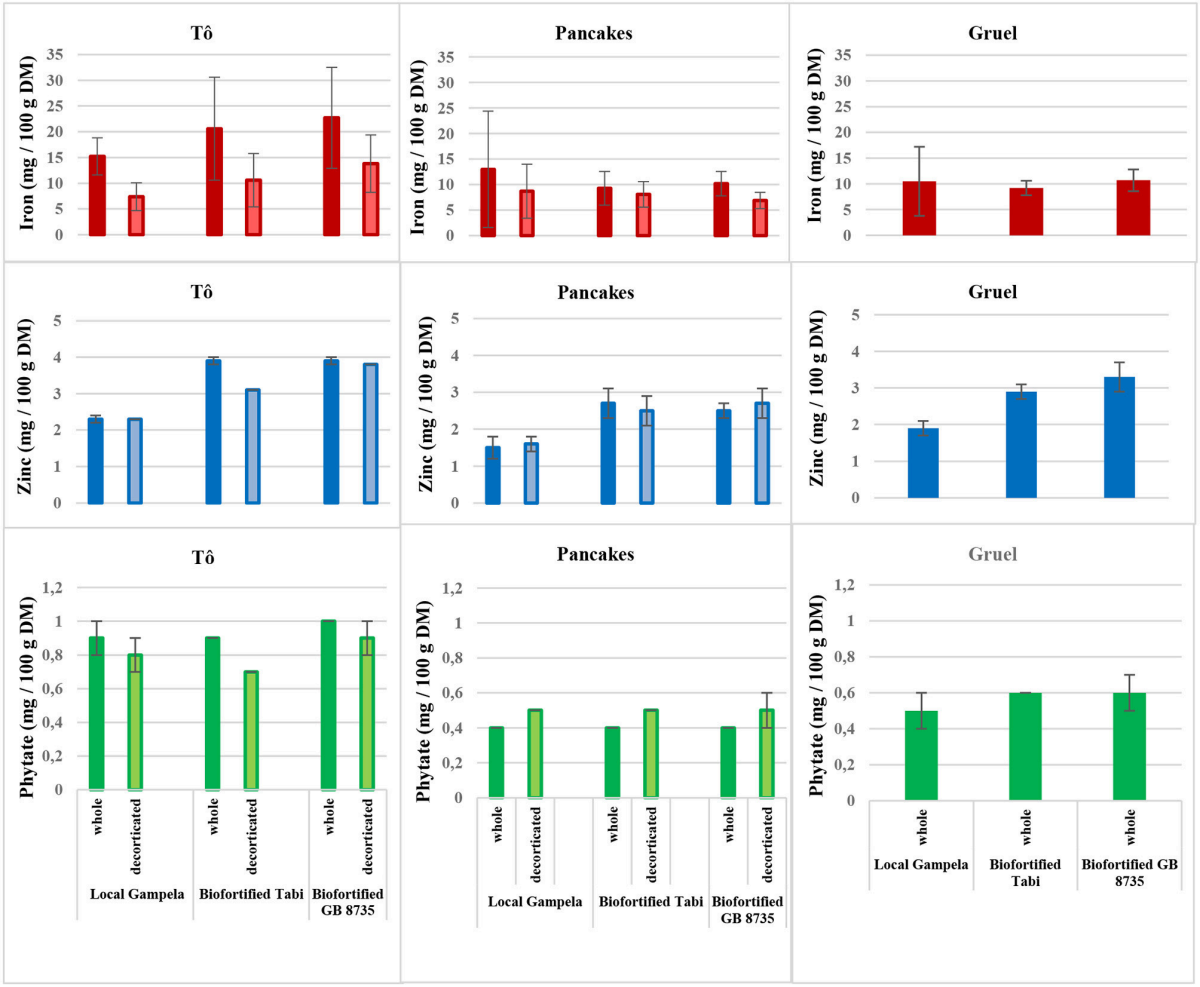
Iron, Zinc, and Phytate contents (mean ± SD, *n* = 3) of dishes (tô, pancakes, and gruel) prepared with different varieties of local and biofortified millet, whole or decorticated (except for gruels, that are traditionally never prepared with decorticated grains).

In prepared dishes, iron content (on a DM basis to enable comparison) varied considerably depending on the dish type and grain variety, but was always higher than in the raw grains, indicating iron contamination during processing (Figure 1). This could originate from contamination of the raw grains by soil because the cooks did not wash the grains as thoroughly as we did in the laboratory. Additional contamination could be due to the use of water traditionally stored in metal tanks. Dust during sun-drying of the grains, if any, and the use of rusty cooking utensils (35–37) are also frequent causes of iron contamination.

Iron content in tô prepared from whole grains was higher than in pancakes and gruel, probably because tô processing is simpler with fewer steps, without mixing and thus dilution with other ingredients. During gruel processing, the first step (soaking the grains) could have contributed to the loss of minerals (38), and also the subsequent step of sieving that results in the removal of the grain peripheral parts that are rich in micronutrients. During pancake processing, iron content could decrease when the flour is sieved or mixed with other ingredients, such as rice or maize. All dishes had much higher iron content when prepared with whole grains than when made with decorticated grains. Indeed, as reported in Hama et al. (32), the removal of the grain pericarp that includes contaminant iron from the soil reduces iron content.

Regardless of the millet variety, the zinc content of the dishes expressed on a DM basis showed few variations linked to processing, probably due to the low zinc content in the environment. Like iron content, and for the same processing reasons, zinc content was higher in tô than in pancakes and gruel. Zinc content was poorly affected by decortication. This confirms previous results (32) showing that little zinc, which is mainly located in the grain aleurone layer and germ, is removed during reasonable decortication because millet germ is embedded in the endosperm. Tô prepared with biofortified millet (decorticated or not) grains had higher iron and zinc contents than when prepared with the local variety.

The phytate content was higher in tô than in pancakes and gruel because grains used for tô do not need to be soaked and sieved. Indeed, phytate content can be reduced during soaking and fermentation due to activation of endogenous or microbial phytases (38). This is the case during gruel and pancake processing.

The phytate-to-iron molar ratios were higher than 1 (Table 3), the threshold proposed for improved predicted absorption (4), although they could have been underestimated due to the probable inclusion of contaminant iron. The phytate-to-zinc molar ratios were higher than 18 for tô, thus predicting low zinc absorption (33), even when prepared with biofortified varieties. However, in pancakes and gruel prepared with biofortified varieties, the phytate-to-zinc molar ratios were around 18 and sometimes lower, suggesting a slightly enhanced zinc absorption.

**Table 3:** Molar ratios of dishes prepared with different varieties of local and biofortified millet.

**Dishes**	**Variety**		DM* (%)	**Phytate-to-Iron molar ratio**	**Phytate-to-Zinc molar ratio**
Tô	Local Gampela	Whole	23.4 ± 3.2	5.0	38.8
		Decorticated	24.2 ± 2.6	9.5	35.7
	Biofortified Tabi	Whole	23.7 ± 3.7	3.9	27.0
		Decorticated	23.2 ± 3.1	5.5	22.4
	Biofortified GB 8735	Whole	24.2 ± 2.6	3.6	24.1
		Decorticated	22.2 ± 3.8	5.5	23.1
Pancakes	Local Gampela	Whole	57.2 ± 5.0	2.8	28.2
		Decorticated	57.3 ± 9.2	5.1	31.1
	Biofortified Tabi	Whole	55.6 ± 1.2	4.0	16.1
		Decorticated	54.8 ± 5.7	5.1	19.1
	Biofortified GB 8735	Whole	55.8 ± 4.6	3.7	17.3
		Decorticated	54.2 ± 3.8	6.4	19.1
Gruel	Local Gampela	Whole	9.2 ± 4.7	4.2	27.3
	Biofortified Tabi	Whole	10.1 ± 4.9	5.4	20.2
	Biofortified GB 8735	Whole	9.4 ± 4.8	4.4	16.9

* *DM: Dry Matter*.

### Potential Contribution of Biofortified Millet-Based Tô to the Iron and Zinc Requirements of Young Children

The potential contribution of millet-based tô to the iron and zinc Recommended Nutrient Intakes (RNI) (39) was then calculated, using low bioavailability values (5% for iron and 15% for zinc), due to phytate-to-mineral molar ratios above 1 for iron and above 18 for zinc (Table 4).

**Table 4:** Contribution to the daily iron and zinc requirement^*^ of a portion of tô prepared with local or biofortified millet varieties, decorticated or not, considering a low bioavailability for iron (5%) and zinc (15%).

Contribution to the mineral requirements*(%)		**Age group**			
		**6-11 months**		**12–23 months**	
		**Whole**	**Decorticated**	**Whole**	**Decorticated**
Mean portion of tô consumed g/meal^30^	on wet basis	76		126	
	on DM basis	15		25	
	on energy basis**	17.1		15.4	
IRON***					
	Local Gampela	12.3/**2.3**	6.0/**1.9**	33.3/**6.4**	16.2/**5.0**
	Biofortified Tabi	16.6/**5.2**	8.5/**3.1**	45.1/**14.2**	23.4/**8.5**
	Biofortified GB 8735	18.4/**4.3**	11.1/**4.6**	49.9/**11.6**	30.2/**12.5**
**ZINC**					
	Local Gampela	4.1	4.1	7.0	7.0
	Biofortified Tabi	6.3	5.5	10.7	9.4
	Biofortified GB 8735	7.0	6.8	11.9	11.6

*Bold values correspond to contribution to mineral requirements calculated with the tô paste without contamination*.

(*) These are the Recommended Nutrient Intakes (39) expressed in bioavailable minerals, i.e., for iron, 0.93 mg for 6–11 month-old infants and 0.58 mg for the 12–23 month old children; for zinc, 1.26 mg for 6–11 month-old infants, and 1.25 mg for the 12–23 month-old children;

(**) calculated using energy requirements from complementary food for young children (40);

(***) *The contribution to requirements was calculated using data obtained with home-made tô (i.e., with iron contamination, Table 3) or with laboratory-made tô (**no expected iron contamination**), respectively 2.7, 6.7, and 5.3 g /100 g of iron on DM basis for tô made from whole Local Gampela, Biofortified Tabi and Biofortified GB 8735, and respectively 2.0, 6.0, and 4.0 g/100 g of iron on DM basis for tô made from decorticated Local Gampela, Biofortified Tabi and Biofortified GB 8735*.

The portions of tô used in our calculations (15 and 25 g DM per meal for children aged 6–11 and 12–23 months, respectively) came from a food consumption survey of Burkinabé children (41). Results were calculated by taking into account the iron content of tô listed in Table 3, but also the iron content of tô prepared with the same varieties, but in the laboratory (rather than home-made) where grains were carefully washed and decorticated to eliminate contaminant iron (26). Tô made with whole biofortified grains “with iron contamination” (i.e., home-made) contributed to almost 50% of the iron requirements of 12–23-month-old children. However, contaminant iron is not as bioavailable as intrinsic iron (37), and using contaminant iron data could lead to a marked overestimation of tô contribution to iron requirements. The calculations obtained with data “without contamination” seemed more realistic: for 6–11 month-old children, they were low, below 5%, regardless of the variety and the degree of decortication, due to the small intakes and the very high RNI in this age group. For the 12–23 month-old children, the iron contribution of dishes prepared with biofortified millet varieties was double compared with the local variety, reaching about 14%. After decortication, the contribution to the iron RNI of tô prepared with local Gampela or biofortified Tabi millet decreased, while that of tô prepared with biofortified GB 8735 remained unchanged. This could be explained by GB 8735's grain hardness that contributed to higher yield and then lower iron losses during decortication. The contribution of biofortified millet varieties to zinc daily requirements was higher than that of the local variety, but remained too low to satisfy the RNI. This shows the need to concomitantly implement biofortification and diversification strategies toward zinc-rich foods, such as animal products.

## Conclusion

Although dishes prepared with biofortified millet were generally well-accepted (from “neither pleasant nor unpleasant” to “pleasant”), the three dishes prepared with the local millet variety were preferred, and these preferences were statistically relevant. Thus, improvement of some sensory parameters of the biofortified millet varieties used in this study, such as color or processing changes, would be advisable. The estimated contributions to iron and zinc requirements of one portion of tô prepared with the biofortified millet varieties used in this study were higher (1.5–2 times) than those of a portion of tô made with the local millet, although they were still quite low. As in Ouagadougou cereals are eaten in different forms by young children several times per day, the use of biofortified millet varieties could have a positive effect on iron and zinc intakes in the long term. Associated with nutritional education and combined with food diversification, millet biofortification could help to improve the supply of iron and zinc in the monotonous diets of low-income people in West-Africa. Moreover, this would support sustainable strategies against malnutrition because the use of the seeds of these biofortified varieties obtained by conventional breeding does not require special regulations and would not replace, but rather complement local varieties, provided they are easily available at competitive prices to farmers.

## Ethics Statement

Our study was exempt from ethics approval according to national legislation and institutional guidelines.

## Author Contributions

CM-R and CI-V: conceptualization, formal analysis, and project administration. FH-B, CM-R, and CI-V: methodology. FH-B: investigation. CI-V: writing—original draft preparation. CM-R, EW, and CI-V: writing review and editing. CI-V: visualization. BD, EW, CM-R, and CI-V: supervision. CM-R: funding acquisition.

### Conflict of Interest Statement

The authors declare that the research was conducted in the absence of any commercial or financial relationships that could be construed as a potential conflict of interest.
